# *Ajania pacifica* (Nakai) K. Bremer and Humphries Extract Limits MYC Expression to Induce Apoptosis in Diffuse Large B Cell Lymphoma

**DOI:** 10.3390/cimb46050278

**Published:** 2024-05-11

**Authors:** Ye-Rin Woo, Chan-Seong Kwon, Ji-Eun Lee, Byeol-Eun Jeon, Tae-Jin Kim, Joy Choo, Young-Seob Seo, Sang-Woo Kim

**Affiliations:** 1 Department of Integrated Biological Science, College of Natural Sciences, Pusan National University, Busan 46241, Republic of Korea; sgate123@pusan.ac.kr (Y.-R.W.); ckstjd5091@naver.com (C.-S.K.); dlwldms4535@naver.com (J.-E.L.); starsilver20@naver.com (B.-E.J.); tjkim77@pusan.ac.kr (T.-J.K.); 2 Department of Biological Sciences, College of Arts and Sciences, Texas Tech University, Lubbock, TX 79409, USA; j.arirose01@gmail.com; 3 Korea Research Institute of Standard and Science, Daejeon 34113, Republic of Korea; yseo@kriss.re.kr; 4 Department of Biological Sciences, College of Natural Sciences, Pusan National University, Busan 46241, Republic of Korea

**Keywords:** *Ajania pacifica* (Nakai) K. Bremer and Humphries, anti-cancer activities, B-cell lymphoma, MYC expression

## Abstract

The proto-oncogene MYC is frequently dysregulated in patients with diffuse large B-cell lymphoma (DLBCL) and plays a critical role in disease progression. To improve the clinical outcomes of patients with DLBCL, the development of strategies to target MYC is crucial. The use of medicinal plants for developing anticancer drugs has garnered considerable attention owing to their diverse mechanisms of action. In this study, 100 plant extracts of flora from the Republic of Korea were screened to search for novel agents with anti-DLBCL effects. Among them, *Ajania pacifica* (Nakai) K. Bremer and Humphries extract (APKH) efficiently suppressed the survival of DLBCL cells, while showing minimal toxicity toward normal murine bone marrow cells. APKH suppressed the expression of anti-apoptotic BCL2 family members, causing an imbalance between the pro-apoptotic and anti-apoptotic BCL2 members. This disrupted mitochondrial membrane potential, cytochrome c release, and pro-caspase-3 activation and eventually led to DLBCL cell death. Importantly, MYC expression was markedly downregulated by APKH and ectopic expression of MYC in DLBCL cells abolished the pro-apoptotic effects of APKH. These results demonstrate that APKH exerts anti-DLBCL effects by inhibiting MYC expression. Moreover, when combined with doxorubicin, an essential component of the CHOP regimen (cyclophosphamide, doxorubicin, vincristine, and prednisone), APKH synergistically enhanced the therapeutic effect of doxorubicin. This indicates that APKH may overcome drug resistance, which is common in patients with refractory/relapsed DLBCL. To identify compounds with anti-DLBCL activities in APKH, the chemical profile analysis of APKH was performed using UPLC-QTOF/MS^e^ analysis and assessed for its anticancer activity. Based on the UPLC-QTOF/MS^e^ chemical profiling, it is conceivable that APKH may serve as a novel agent targeting MYC and sensitizing drug-resistant DLBCL cells to CHOP chemotherapy. Further studies to elucidate how the compounds in APKH exert tumor-suppressive role in DLBCL are warranted.

## 1. Introduction

Diffuse large B-cell lymphoma (DLBCL) is the most prevalent aggressive non-Hodgkin lymphoma, accounting for 30% of all lymphoma cases in Western countries [1]. Molecular categorization of DLBCL, based on gene expression analysis, has identified various subtypes of this malignancy. Genetic studies have established that germinal center B-cell-like (GCB) and activated B-cell (ABC) subtypes are pathogenetically distinct [2]. The GCB and ABC subtypes originate from discrete cells. GCB-like DLBCLs express genes typical of germinal center B-cells and have a good prognosis, while ABC-like DLBCLs have a poor prognosis and express genes in activated peripheral B-cells [3].

Traditionally, chemoimmunotherapy with R-CHOP (rituximab, cyclophosphamide, doxorubicin, vincristine, and prednisone) has been a cornerstone in the treatment of DLBCL, resulting in long-term and disease-free survival for approximately 60% of patients [4]. However, this narrative evolves significantly when considering the management of relapsed or refractory cases. Previously perceived as having a dismal prognosis with cure rates around 10%, recent advancements in therapeutic strategies have markedly improved outcomes [5]. The advent of CAR-T cell therapy has revolutionized the treatment landscape for relapsed DLBCL patients, with cure rates now approaching 40%. Furthermore, bispecific antibodies are poised to offer a comparable cure rate across the spectrum of relapsed patients, signaling a new era of hope and efficacy in the treatment of this challenging condition [6].

Despite these advancements, the quest for novel therapeutic options remains imperative. Researchers in the last decades have studied several therapeutic plants and herbs owing to their diverse biological properties. This trend emphasizes the role of nature as a source of potent agents capable of combating complex diseases. Derived from the Madagascar periwinkle, vincristine’s success in the R-CHOP regimen exemplifies the therapeutic potential of plant extracts in medical oncology. *Ajania pacifica* (Nakai) K. Bremer and Humphries (APKH) is native to the coastal areas of Asia, including Japan and Korea. It has been used in Asian traditional medicine to treat various ailments. However, the antitumorigenic effects of this plant on DLBCL remain unexplored. 

Several growth-stimulating signals are associated with the etiology of DLBCL. The proto-oncogene, MYC, is a major target of these pathways [7,8,9]. Dysregulation of MYC causes lymphomagenesis through the loss of tight control of MYC expression, resulting in overexpression of intact MYC, which differs from somatic mutations or fusion proteins observed in the majority of other oncogenes [10]. Notably, MYC rearrangement is frequently observed in DLBCL with a complex karyotype. The negative prognosis associated with MYC rearrangement is mostly attributed to concomitant BCL2 apoptosis regulator or BCL6 rearrangements [11]. Approximately 30% of GCB-like subgroup cases and < 5% of ABC-like DLBCL cases exhibit a BCL2 rearrangement. Regardless of the International Prognostic Index and BCL2 levels, high MYC expression is associated with decreased overall survival. When paired with BCL2 expression, the predictive value of MYC expression increases. Approximately 20–35% of cases of DLBCLs exhibit co-expression of MYC and BCL2. These DLBCLs are referred to as “double-expressor lymphomas” and the vast majority do not exhibit contemporaneous rearrangements of MYC and BCL2 [12,13].

The mitochondrial mechanism of apoptosis is controlled by several BCL2 family members, which are categorized into three groups: the pro-apoptotic multidomain, anti-apoptotic multi-domain, and BH3-only proapoptotic proteins [14].

In this study, we demonstrated the in vitro activities of APKH extract against DLBCL cell lines and its underlying mechanism of action. Our results showed that APKH extract significantly downregulated the expression of the oncogene MYC, leading to the induction of DLBCL cell death. Therefore, APKH may be a viable natural product for developing innovative therapeutics for B-cell lymphoma.

## 2. Materials and Methods

### 2.1. Material and Preparation of Extracts

Entire plants of APKH, which belongs to the family Compositae, were harvested from Jeju Island in the Republic of Korea and gathered from the Korea Plant Extract Bank of Korea Research Institute of Bioscience and Biotechnology (KRIBB, PE2357.9), ensuring the use of whole plants in our research. The plant extracts were produced using 95% ethyl alcohol. The institute maintained a collection of voucher specimens. After dissolving the fine powder (20 g) with dimethyl sulfoxide (200 μL), the resulting extract was kept at 4 °C.

### 2.2. Cell Culture and Antibodies

Human DLBCL cell lines (Ly1, DHL6, and U2932) were used for all assays and the characteristics of these DLBCL cell lines are listed in Supplementary Table S1. Cells were maintained in RPMI-1640 medium (Hyclone, Logan, UT, USA) supplemented with 10% fetal bovine serum (FBS; Hyclone, Melbourne, VIC, Australia), 1% L-glutamine, 1% N-2-hydroxyethylpiperazine-N’-2-ethanesulfonic acid (HEPES) buffer, and 1% penicillin/streptomycin. Cells were cultured in a humidified atmosphere at 37 °C under 5% CO_2_ within an incubator. The Ly1, DHL6, and U2932 DLBCL cell lines were a generous gift from Dr Ricardo Aguiar (University of Texas Health Science Center at San Antonio, San Antonio, TX, USA). For cell line authentication, short tandem repeat (STR) analysis was performed by Cosmogenetech (Seoul, Republic of Korea) and all cell lines were negative for mycoplasma contamination. Normal bone marrow (BM) cells isolated from wild-type C57BL/6JJmsSIc mice were cultured in RPMI-1640 medium with 10% FBS (Hyclone).

Primary antibodies used for western blots included anti-4EBP1 (1:1000 dilution, 9452S; Cell Signaling Technology, Beverly, MA, USA), anti-phospho-4EBP1 (1:1000 dilution, 9459S; Cell Signaling Technology), anti-AKT (1:1000 dilution, 9272S; Cell Signaling Technology), anti-phospho-AKT (1:1000 dilution, 9271S; Cell Signaling Technology), anti-BCL2 (1:1000 dilution, sc-7381; Santa Cruz Biotechnology, Dallas, TX, USA), anti-BCL2L1 (1:1000 dilution, 14-6994-81; eBioscience, San Diego, CA, USA), anti-PARP-1 (1:2000 dilution, sc-7150; Santa Cruz Biotechnology), anti-pro-caspase-3 (1:2000 dilution, H-277; Santa Cruz Biotechnology), anti-MYC (1:1000 dilution, ab32072; Abcam, Cambridge, UK), and anti-*β*-actin (1:5000 dilution, sc-47778; Santa Cruz Biotechnology) antibodies. Anti-mouse and anti-rabbit secondary antibodies conjugated with horseradish peroxidase (HRP) were purchased from Bethyl Laboratories, Inc. (A120-101p and A90-116p-33, respectively; Montgomery, TX, USA). 

### 2.3. Cell Proliferation Assay

Cell growth was determined using cell counting assay, in which 2.0 × 10^5^ cells/well were seeded in a 24-well cell culture plate (SPL Life Science, Pocheon, Republic of Korea) on day zero and counted every 24 h using a hemocytometer (Marienfeld, Lauda-Königshofen, Germany) under a phase-contrast microscope (Olympus CKX41; Olympus Corporation, Tokyo, Japan).

### 2.4. Trypan Blue Staining

The cytotoxicity of plant extracts was evaluated using trypan blue assay. Briefly, cells were seeded at 1.5 × 10^5^ cells/well in 24-well plates and treated with the plant extracts for 48 and 72 h. Cells were harvested and stained with a 1:1 mixture of trypan blue stain (Sigma; St Louis, MO, USA) diluted to 0.4% for 3 min at room temperature (20–25 °C). Positively labeled cells were tailed using a hemocytometer under a phase-contrast microscope. Cytotoxicity was calculated as follows:

Percent cytotoxicity = [(total cells − viable cells)/total cells] × 100. (1)

### 2.5. Mitochondrial Membrane Potential (MMP)

MMP was assessed using florescence microscopy following the treatment of Ly1 with 100 μg/mL APKH for 24 h and incubation with 5,5′,6,6′-tetrachloro-1,1′,3,3′-tetraethylben zimidazolylcarbocyanine iodide (JC-1; Abnova, Taipei, Taiwan) for 15 min at 37 °C.

### 2.6. Measurement of Cell Viability and Apoptosis Assays

To determine cell viability, CellTiter 96 AQueous One Solution [3-(4,5-dimethylthiazol-2-yl)-5-(3-carboxymethoxyphenyl)-2] cell proliferation assays (MTS) were performed according to the manufacturer’s instructions (Promega, Madison, WI, USA) after Ly1, DHL6 or BM cells were treated with plant extracts. Briefly, 3.0 × 10^4^ cells/well in RPMI1640 containing 10% FBS were seeded in 96-well-plates and treated for 24 h, and cell viability was measured using MTS assay. The MTS reagent (30 μL) was added to each well and incubated at 37 °C for 4 h. Absorbance was measured at 450 nm using a GloMax^TM^ Microplate multi-mode reader (Promega). 

Ly1 cells were seeded at a density of 1.0 × 10^6^ cells/well in 12-well plates and treated with APKH for 24 h. After staining with propidium iodide (PI; BD Biosciences, Franklin, NJ, USA), the rate of apoptosis was evaluated using flow cytometry (FACSVerse; BD Biosciences).

### 2.7. Western Blot Analysis

Western blotting was performed to determine the expression of apoptosis-associated proteins in Ly1 cells. Ly1 cells were seeded in a 12-well plate at 2.0 × 10^6^ cells/well and treated for 24 h with APKH. Cells were then harvested and lysed in RIPA buffer (ELPIS Biotechnology, EBA-1149, Daejeon, Republic of Korea) with 1 mM Na-vanadate (Sigma Aldrich, St. Louis, MO, USA), 50 mM *β*-glycerophosphate disodium salt (G9422; Sigma Aldrich), 142 mM *β*-mercaptomethanol (41300000-1; BioWORLD, Visalia, CA, USA), ProteaseArrest^TM^ (G-Bioscience; Maryland Heights, MO, USA), and 5 mM ethylenediaminetetraacetic acid (EDTA; G-Bioscience). Protein samples were heated at 100 °C for 10 min in 1× sample buffer and separated using sodium dodecyl sulfate–polyacrylamide gel electrophoresis. Thereafter, the separated proteins were loaded onto Immobilon-P Transfer membranes, which were then blocked in 1% bovine serum albumin (BSA; MP Biomedicals, Santa Ana, CA, USA) dissolved in Tris-buffered saline containing 0.1% Tween-20 (TBST).

Membranes were incubated with primary antibodies for 16 h at 4 °C on a rotor and washed thrice for 5 min each with TBST. The membranes were incubated with anti-mouse secondary antibodies or anti-rabbit secondary antibodies at room temperature for 1 h and rinsed thrice for 10 min each. Protein bands on membranes were treated with a chemiluminescent substrate (EzWestLumi plus (ATTO, Osaka, Japan)) and visualized using a Luminograph II system (ATTO, Osaka, Japan).

### 2.8. RNA Isolation and Quantitative Real-Time RT-PCR (qRT-PCR)

RNA was extracted using TRIzol reagent (FATRR 001; Favorgen, Wien, Austria) and transcribed into cDNA using a Prime-Script RT Reagent Kit following the manufacturer’s instructions (RR047A; Takara, Kusatsu-shi, Japan). The transcription levels of MYC, BCL2, and BCL2L1 were measured by qRT-PCR using TOPreal qPCR PreMIX SYBR Green with low ROX (RT500M; Enzynomics, Daejeon, Republic of Korea). PCR was performed as follows: 95 °C for 15 min, 40 cycles at 95 °C for 15 s, 59 °C for 30 s, and 72 °C for 30 s. The following primer sequences were used: 

BCL2 Forward: 5′-GGATGCCTTTGTGGAACTGT-3′ 

BCL2 Reverse: 5′-AGCCTGCAGCTTTGTTTCAT-3′ 

BCL2L1 Forward: 5′-GGCTGGGATACTTTTGTGGA-3′

BCL2L1 Reverse: 5′-GGGAGGGTAGAGTGGATGGT-3′ 

MYC Forward: 5′-CTCCTGGCAAAAGGTCAGAG-3′

MYC Reverse: 5′-TCGGTTGTTGCTGATCTGTC-3′

TBP Forward: 5′-TATAATCCCAAGCGGTTTGCTGCG-3′ 

TBP Reverse: 5′-AATTGTTGGTGGGTGAGCACAAGG-3′

### 2.9. Cell Cycle Analysis

DLBCL cells were seeded (1.5 × 10^6^) and treated with APKH for 24 h. Cells were harvested, washed twice with 1× PBS, and fixed overnight in 75% ethanol at 4 °C. After centrifugation (2000 rpm, 5 min, 21 °C), cells were stained for 1 h at room temperature with PI staining solution (40 μg/mL PI in PBS with 0.1% Triton X and 100 μg/mL RNase A) and analyzed using flow cytometry (BD FACSAria^TM^ Fusion Flow Cytometer, BD Biosciences).

### 2.10. Ectopic Expression of MYC in Ly1 Cells

Control Ly1 cells or Ly1 cells stably expressing MYC were generated using pCDH-CMV-MCS-EFI-copGFP lentiviral constructs (Systems Biosciences, Mountain View, CA, USA) as previously described [15] and designated as Ly1 *CDH* or Ly1 *CDH*-*MYC*, respectively.

### 2.11. Ultra-Performance Liquid Chromatography-Ion Mobility Separation-Quadrupole Time-of-Flight/Tandem Mass Spectrometry (UPLC Q-TOF/MS^e^) Analysis

To analyze the components of APKH, the UPLC (AQUITY^TM^ UPLC Class 1, Waters Corp., Milford, MA, USA) system was used. Separation of the physiological compounds was performed on an ACQUITY UPLC BEH C_18_ column (2.1 × 100 mm) at a flow rate of 800 L/h. The mobile phases consisted of solvent A (0.1% formic acid in distilled water) and solvent B (acetonitrile). The gradient conditions were as follows: 0–14 min, 8% B; 14–17 min, 40% B; 17–20 min, 100% B, 20–22 min, 8% B. THE UPLC-QTOF/MS^e^ system was analyzed using MS^e^ data analysis 4.1 software (Waters Masslynx^TM^. Waters Corp.). 

### 2.12. Animal Studies

To examine in vitro toxicity, BM cells were obtained from 9-week-old male wild-type C57BL/6 mice and treated with the plant extracts, followed by MTS assays. The animal experimental protocol used in this study was reviewed and approved by the Institutional Animal Care and Use Committee of Pusan National University (PNU-2021-3056).

### 2.13. Statistical Analysis

All experiments were performed in triplicate to ensure repeatability. Data are presented as mean ± standard deviation (SD). Statistically significant differences were determined by Mann–Whitney U test and one-way analysis of variance (ANOVA) with Tukey’s post hoc test using Microsoft Office Excel and GraphPad Prism 5.03 software (GraphPad Software, Inc., San Diego, CA, USA).

## 3. Results

### 3.1. Cytotoxicity of APKH Extract on DLBCL Cells

**Figure 1:**
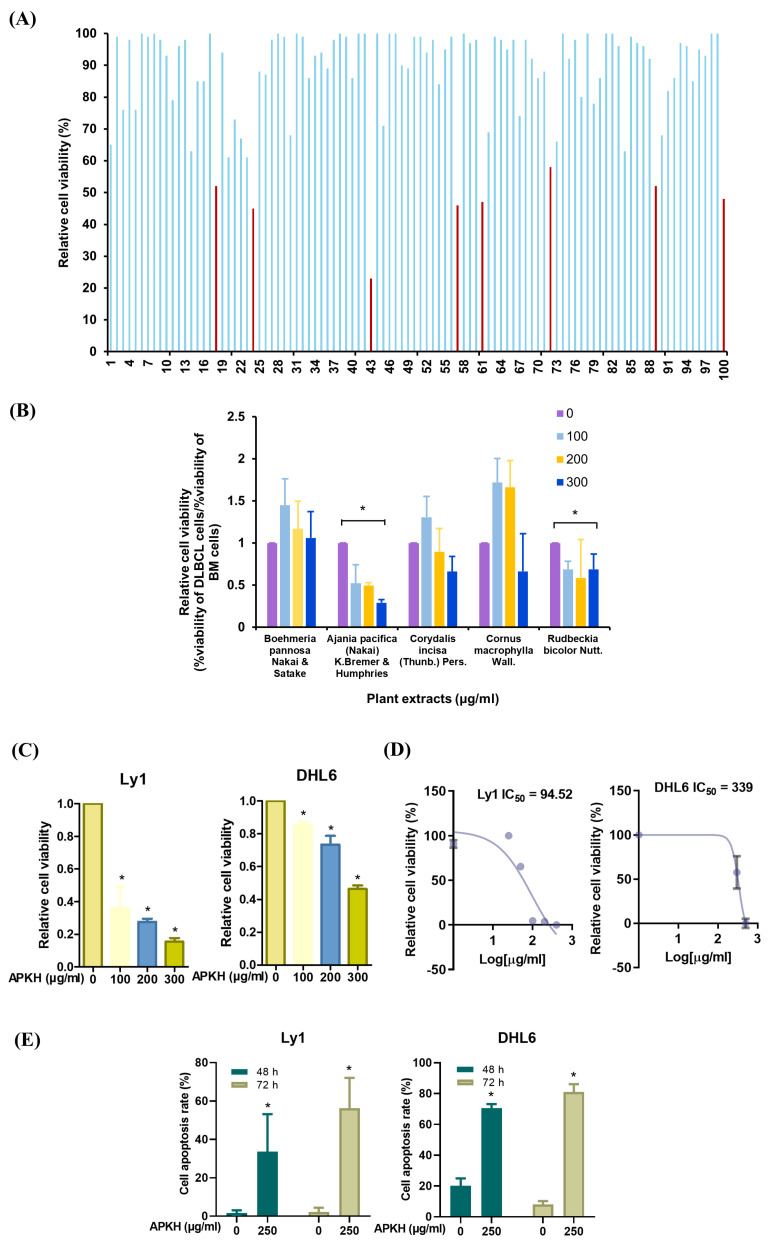

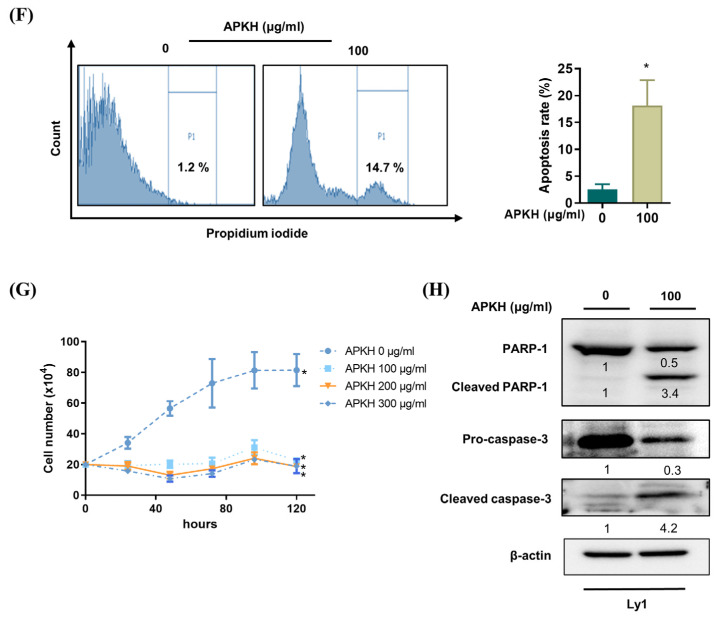
APKH triggers apoptosis in DLBCL. (**A**) Ly1 cells were treated with extracts of 100 species of plants for 72 h, and the cytotoxicity was analyzed by trypan blue assays. The red bars represent cytotoxicity of 40% or higher. The scientific names of corresponding plant extracts are listed in Supplementary Table S2. (**B**) DLBCL Ly1 or normal bone marrow (BM) cells were exposed to APKH (0, 100, 200, or 300 μg/mL) for 24 h, and MTS assay was performed to measure cell viability. Statistical significance was analyzed using a two-tailed Student’s *t*-test (* *p* < 0.05). (**C**) Ly1 and DHL6 cells were treated with APKH (0, 100, 200, or 300 μg/mL) for 24 h, and MTS assay was performed (* *p* < 0.05). (**D**) The IC_50_ values of APKH in Ly1 and DHL6 cells were calculated using GraphPad Prism v.5 software. (**E**) Ly1 and DHL6 cells were treated with APKH (250 μg/mL) for 48 or 72 h. Cell number was measured using trypan blue assay (* *p* < 0.05). (**F**) Ly1 cells were treated with APKH (250 μg/mL) for 24 h and then stained with PI and analyzed by flow cytometry to measure apoptotic rates (* *p* < 0.05). (**G**) Cell proliferation was analyzed by cell counting (* *p* < 0.05). (**H**) Following treatment with APKH, Western blot analysis was performed to detect changes in PARP-1 and pro-caspase 3 levels. β-actin was used as a loading control. The numbers presented beneath each image represent a semi-quantitative evaluation of every line.

We conducted a screening of 100 distinct extracts for their effects on the viability of Ly1 human DLBCL cells using trypan blue (Supplementary Table S2). Eight extracts (*Boehmeria pannosa* Nakai and Satake, APKH, *Corydalis incisa* (Thunb.) Pers., *Fatsia japonica* (Thunb.) Decne. and Planch., *Isodon inflexus* (Thunb.) Kudo, *Ribes fasciculatum* var. *chinense* Maxim., *Cornus macrophylla* Wall., and *Rudbeckia bicolor* Nutt. extracts) reduced the viability of DLBCL cells by >40% (Figure 1A). Despite their significant cytotoxicity, we excluded certain extracts (*Fatsia japonica* (Thunb.) Decne. and Planch., *Isodon inflexus* (Thunb.) Kudo, and *Ribes fasciculatum* var. *chinense* Maxim. extracts) from our analysis based on the results of previous studies that reported high toxicity in normal murine BM [16]. Subsequently, we compared the cytotoxic effects of these extracts on Ly1 and BM cells. APKH showed selective cytotoxicity toward Ly1 cells with no significant effect on BM cells (Figure 1B and Figure S1). We conducted further experiments using two additional cell lines to ascertain the specificity of APKH’s effect: DHL6, representing the GCB subtype, and U2932, representing the ABC subtype of DLBCL. Similar to our findings in Ly1 cells, we observed a significant reduction in the viability of DHL6 cells upon APKH treatment, whereas U2932 cells showed no such reduction (Figure 1C and Figure S2). This raises the possibility that APKH’s pro-apoptotic effect might be specific to the GCB subtype of DLBCL, a hypothesis that warrants additional investigation. The half-maximal inhibitory concentration (*IC*_50_) of APKH was 94.52 μg/mL in Ly1 cells and 339 μg/mL in DHL6 cells (Figure 1D). Based on these findings, we selected APKH for further investigation as a potential cytotoxic agent against DLBCL. APKH was concluded to be a potential cytotoxic agent against DLBCL after conducting multiple assays, such as trypan blue assays, PI staining followed by flow cytometry analysis, and cell counting. (Figure 1E–G). Consistently, Western blot analysis showed that poly (ADP-ribose) polymerase 1 (PARP-1) and pro-caspase-3 levels decreased, whereas the expression of the cleaved forms of these molecules increased, indicating efficient cell death induced by APKH (Figure 1H). Given that APKH did not arrest cell cycle progression in Ly1 DLBCL cells, the cytotoxic effect of APKH may be mainly due to the induction of cell death (Figure S3). These results demonstrate that APKH could be a potential therapeutic agent for treating DLBCL.

### 3.2. MYC Is an Important Regulator of APKH-Induced Apoptosis

**Figure 2:**
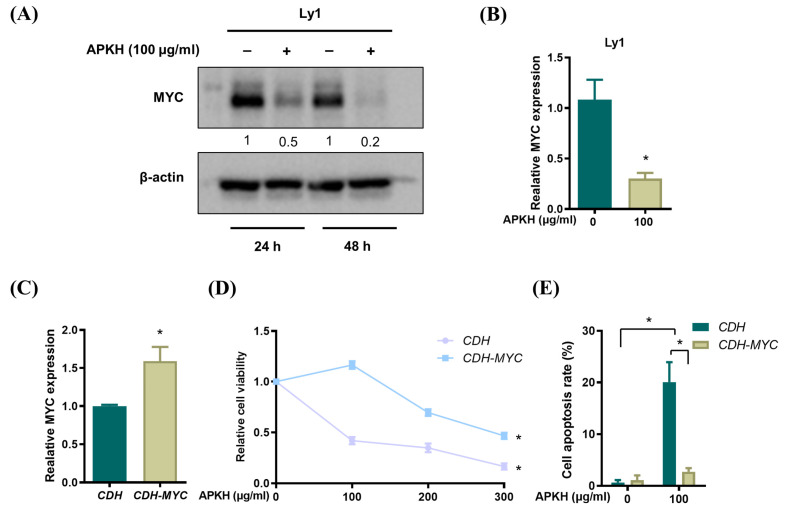
APKH induces apoptosis in DLBCL cell lines by inhibiting MYC. (**A**) Western blot analysis of MYC in Ly1 cells treated with 100 μg/mL APKH for 24 or 48 h. The numbers presented beneath each image represent a semi-quantitative evaluation of every line. (**B**) Real time RT-qPCR was performed to examine MYC mRNA levels in Ly1 cells treated with 100 ug/mL APKH for 48 h (* *p* < 0.05). (**C**) Ly1-*CDH* and Ly1-*CDH-Myc* cells were cultured in the presence of APKH (100 μg/mL) for 24 h, and the mRNA expression of MYC was analyzed by qRT-PCR (* *p* < 0.05). (**D**) Ly1-*CDH* and Ly1-*CDH-Myc* cells were exposed to increasing concentrations of APKH for 24 h, and cell viability was measured by MTS assay. Statistical significance was analyzed using a two-tailed one-way ANOVA (* *p* < 0.05). (**E**) Ly1 cells were treated with APKH (100 μg/mL) for 48 h. Cell number was measured using trypan blue assay (* *p* < 0.05).

MYC dysregulation is frequently found in DLBCL and is associated with poor prognosis. In our previous report, it has been shown that MYC is overexpressed in DLBCL cell lines, including Ly1 and DHL6, and MYC suppression by JQ1 (a potent and specific BRD4 inhibitor) and 10058-F4 (a MYC-Max hetero-dimerization inhibitor) efficiently induced apoptosis in these cells, suggesting that MYC plays a critical role in survival of DLBCL cells [15]. Given these previous results, we hypothesized that APKH might ameliorate MYC expression to induce apoptosis in these cells. To directly investigate whether APKH-induced cell death in DLBCL was associated with MYC downregulation, we exposed Ly1 cells to APKH and analyzed MYC expression using Western blotting. MYC expression was significantly inhibited by APKH, suggesting that MYC is potentially involved in APKH-induced apoptosis (Figure 2A). Consistently, MYC mRNA level was repressed by APKH (Figure 2B). To test whether MYC plays a direct role in this process, we used Ly1 cells ectopically expressing MYC, as characterized in our previous study [17]. Predictably, Ly1 cells transduced with lentiviral *CDH-MYC* vector (Ly1-*CDH-MYC*) exhibited markedly higher mRNA levels of MYC than those transduced with lentiviral CDH control vector (Ly1-*CDH*) (Figure 2C). This overexpression of MYC rendered Ly1 cells more resistant to the cytotoxic effect of APKH than the control cells, as evidenced by cell viability (Figure 2D). Moreover, Ly1-*CDH-MYC* cells were markedly less sensitive to apoptosis induction by APKH treatment (Figure 2E). Together, these data indicate that inhibition of MYC expression results in APKH-triggered apoptosis.

### 3.3. APKH Regulates Myc Expression via Protein Kinase B (AKT)/Mammalian Target of Rapamycin (mTOR) Signaling

**Figure 3:**
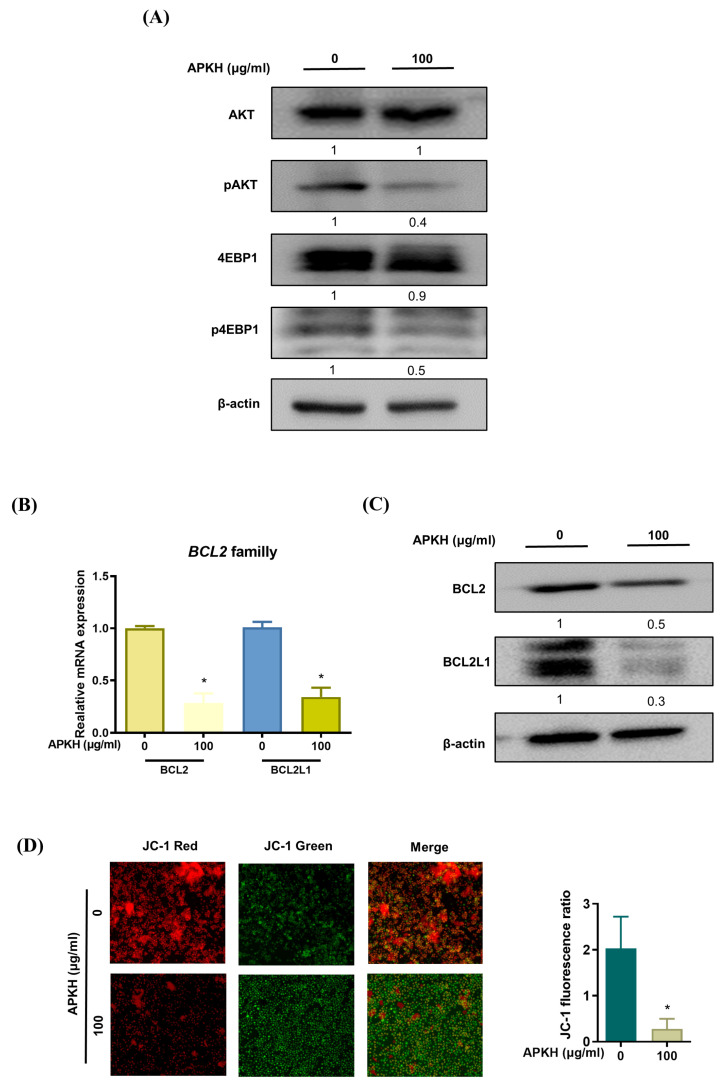
APKH suppresses the expression of anti-apoptotic proteins and disrupts MMP. (**A**) Western blot analysis of phosphorylated and total forms of AKT and 4EBP1 in Ly1 cells treated with APKH for 24 h. (**B**) Ly1 cells were cultured with APKH (100 μg/mL) for 24 h, and the mRNA expression of BCL2 family members was analyzed by qRT-PCR (* *p* < 0.05). (**C**) Western blot analysis of BCL2 and BCL2L1 in Ly1 cells treated with APKH for 24 h. (**D**) Ly1 cells were cultured with APKH (100 μg/mL) for 24 h, stained with JC-1, and observed using a fluorescence microscope at 400× magnification (* *p* < 0.05).

We examined the upstream regulators of MYC expression. The *phosphoinositide−3−kinase*/*AKT*/*mTOR* signaling pathway is critical to tumor development and metastasis and important in MYC regulation. To directly elucidate the involvement of this pathway in APKH-triggered modulation of MYC levels, we treated Ly1 cells with APKH and performed Western blot analysis. APKH efficiently inhibited the phosphorylation of AKT and eukaryotic translation initiation factor 4E (eIF4E)-binding protein 1 (4EBP1), an important downstream target of mTOR signaling, suggesting that APKH suppresses MYC expression through AKT/mTOR signaling (Figure 3A). These results are consistent with our previous reports showing that roflumilast, a phosphodiesterase 4 inhibitor approved by the US Food and Drug Administration, downregulates MYC expression by suppressing AKT/mTOR signaling in DLBCL cells [18], suggesting that the AKT/mTOR pathway is important in the regulation of MYC expression.

### 3.4. APKH Disrupts MMP by Downregulating Anti-Apoptotic BCL2 Genes

Next, we identified the downstream targets of MYC. Interestingly, APKH increased the levels of the cleaved/active form of caspase-3, an executioner caspase involved in apoptosis. This implies an unbalanced ratio between pro-apoptotic and anti-apoptotic BCL2 family members, disruption of the integrity of the outer mitochondrial membrane, cytochrome c release into the cytoplasm, and formation of an apoptosome, leading to the activation of caspases, and ultimately, cell death. Given that anti-apoptotic BCL2 family members are well-established as targets of MYC and that APKH inhibits MYC expression, we speculated that APKH exerts a pro-apoptotic effect by repressing MYC, resulting in the downregulation of anti-apoptotic BCL2 family members. The mRNA levels of BCL2 and BCL2L1 were markedly reduced. (Figure 3B). Our Western blot analysis corroborated these findings, showing a marked reduction in the protein expression of BCL2 and BCL2L1 following APKH treatment (Figure 3C). Therefore, we investigated whether APKH-induced repression of anti-apoptotic BCL2 family members disrupts MMP. Fluorescence microscopic analysis indicated decreased MMP, suggesting that APKH induced apoptosis in DLBCL cells by disrupting MMP and activating caspases (Figure 3D).

### 3.5. APKH Enhances the Cytotoxic Effect of Doxorubicin

Approximately 75% of DLBCL patients exhibit complete response to R-CHOP, a standard chemotherapy for patients with DLBCL; the remaining patients die of the disease mainly owing to drug resistance, thereby necessitating strategies for re-sensitizing drug-resistant DLBCL cells. 

**Figure 4:**
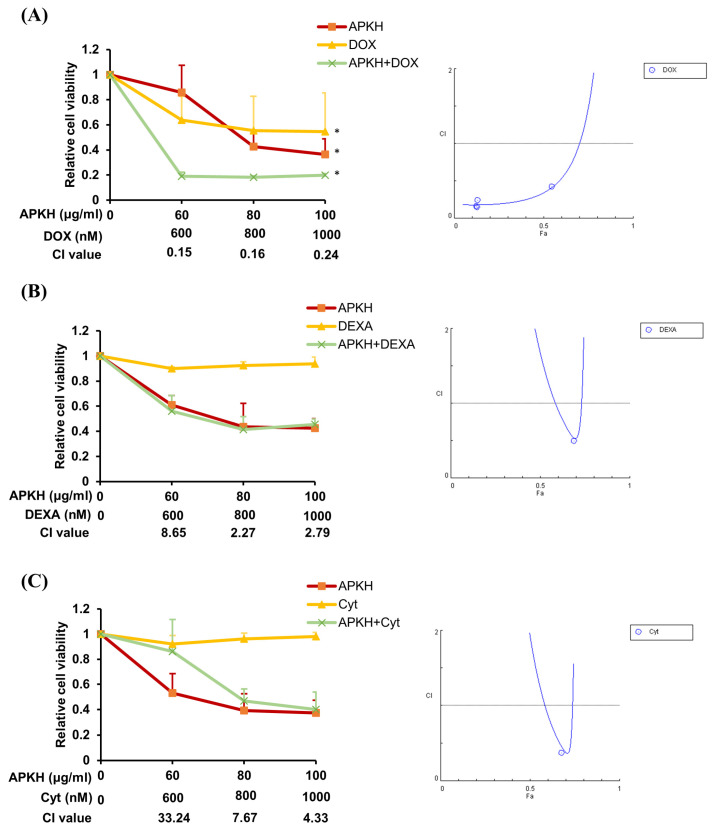
APKH synergizes with doxorubicin to enhance the apoptotic rate in DLBCL cells. (**A**) Ly1 cells were treated with doxorubicin (0–1000 nM) and/or APKH (0–100 μg/mL) for 24 h and an MTS assay was performed to measure cell viability. CI values were calculated using CompuSyn 1.0 software to determine the synergistic effect of APKH and doxorubicin on the viability of Ly1 cells by comparing the effects of combination and single-drug treatments. The synergy levels are as follows: <0.1, very strong synergism; 0.1–0.3, strong synergism; 0.3–0.7, synergism; 0.7–0.85, moderate synergism; 0.85–0.90, slight synergism; 0.90–1.10, nearly additive; 1.10–1.20, slight antagonism; 1.20–1.45, moderate antagonism; and 1.45–3.30, antagonism. * *p* < 0.05. (**B**) Ly1 cells were treated with dexamethasone (0–1000 nM) and/or APKH (0–100 μg/mL) for 24 h and an MTS assay was performed to measure cell viability. (**C**) Ly1 cells were treated with cytarabine (0–1000 nM) and/or APKH (0–100 μg/mL) for 24 h and an MTS assay was performed to measure cell viability.

We investigated whether APKH increases the cytotoxic effects of commonly used chemotherapeutic agents, such as doxorubicin, dexamethasone (a synthetic glucocorticoid), and cytarabine. Co-administration of APKH and doxorubicin exhibited a synergistic cytotoxic effect on Ly1 cells, whereas combined treatment with APKH and doxorubicin exhibited strong to very strong synergy based on the combination index (CI) values (Figure 4A). However, no synergistic effect was observed when APKH was co-administered with dexamethasone or cytarabine (Figure 4B,C). Given that doxorubicin is an important component of R-CHOP, these results suggest that APKH enhances the therapeutic efficacy of doxorubicin and may potentially be used to overcome resistance to R-CHOP.

### 3.6. Compound Analysis

**Figure 5:**
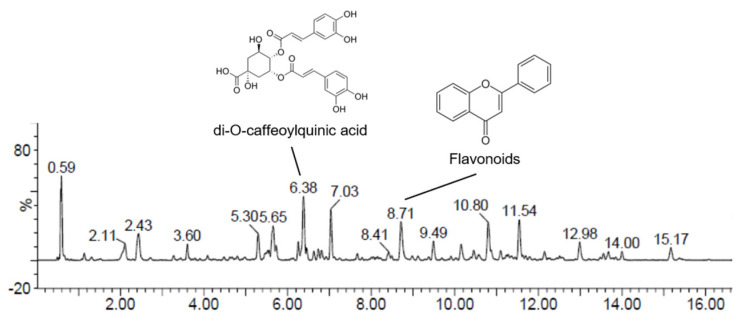
Ultra-performance liquid chromatography-ion mobility separation-quadrupole time-of-flight/tandem mass spectrometry (UPLC Q-TOF/MS^e^) chromatographic profile of APKH.

In our study, we conducted a comprehensive qualitative assessment of the major compounds present in APKH using UPLC-QTOF-MSe analysis (Figure 5). In the total ion current profile of APKH, we detected more than 12 peaks with retention times ranging from 2 to 12 min. Among these peaks, we selected two common ones through the integration of data from negative ion mode, positive ion mode, and UV analyses (Figure S4). These identified compounds, along with their retention times, *m*/*z* values, molecular formulas, and compound names are listed in Table 1.

Of notable significance, the mass spectrometry chromatogram obtained in negative ion mode distinctly disclosed the existence of two remarkable compounds. Compound **1** presented [M − H]^−^ precursor ions at *m*/*z* 515.1186 and was conclusively identified as di-O-caffeoylquinic acid. Compound **2** exhibited [M − H]^−^ precursor ions at *m*/*z* 301.0354 (C_15_H_9_O_7_) and *m*/*z* 285.0404 (C_15_H_9_O_6_), characterizing it as flavonoids [19].

We propose that these compounds, which are commonly found in natural products and known for their diverse bioactivities, such as anti-inflammatory, antioxidative, and anticancer effects, may represent the primary bioactive ingredients in APKH. These bioactive compounds could potentially play a significant role in the anticancer activities observed in DLBCL cells.

## 4. Discussion

In this study, we explored the efficacy of APKH extract in the treatment of Diffuse Large B-Cell Lymphoma (DLBCL), a prevalent and aggressive form of non-Hodgkin lymphoma that often resists current chemoimmunotherapy regimens [20,21]. Our findings illuminate APKH’s potential to mitigate the oncogenic influence of MYC overexpression—a well-documented hallmark of DLBCL pathogenesis associated with adverse outcomes [22]. By demonstrating that DLBCL cells are particularly susceptible to apoptosis upon MYC suppression by APKH, our research underscores the critical role of the MYC pathway in DLBCL’s aggressive behavior and identifies APKH as a potent disruptor of this oncogenic signaling cascade.

**Figure 6:**
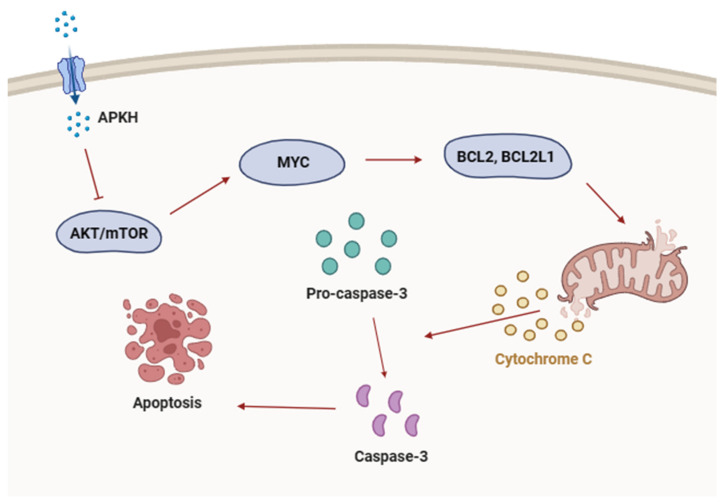
Schematic of APKH-induced cell death. Inhibition of AKT/mTOR activation by APKH suppressed MYC expression, leading to the downregulation of BCL2 and BCL2L1, which induced apoptosis. Unbalanced expression of pro-apoptotic and anti-apoptotic BCL2 family members diminished MMP, resulting in the release of cytochrome c, cleavage/activation of pro-caspase-3, and ultimately cell death.

Apoptosis is a delicately regulated process that is governed by the equilibrium between pro-apoptotic and anti-apoptotic proteins. As depicted in the schematic model (Figure 6), treatment with APKH led to significant alterations in the expression and activation of various molecules implicated in cell survival and apoptosis. First, we analyzed the effect of APKH on the AKT/mTOR signaling pathway, which is essential for cell survival and proliferation [23,24]. APKH effectively suppressed the phosphorylation of AKT and 4EBP1, indicating that APKH inhibits AKT/mTOR signaling. Further analysis revealed APKH’s capability to downregulate MYC expression and alter the balance between pro-apoptotic and anti-apoptotic members of the BCL2 family, favoring apoptosis induction. Notably, APKH’s induction of mitochondrial membrane potential (MMP) disruption and subsequent activation of the caspase cascade signifies a targeted approach to dismantle the intrinsic apoptotic pathway, thereby highlighting its therapeutic promise.

In particular, our study investigated the possibility of combining APKH with standard chemotherapeutic compounds for treating DLBCL. The combination of APKH and doxorubicin exhibited a synergistic cytotoxic effect on DLBCL cells. Therefore, APKH can increase the therapeutic efficacy of doxorubicin, a key component of the R-CHOP regimen, and surmount drug resistance in DLBCL [25]. 

In light of our comprehensive qualitative assessment of the major compounds present in APKH through UPLC-QTOF-MS^e^ analysis, we have identified two prominent compounds, di-O-caffeoylquinic acid and flavonoids, which may play a significant role in APKH’s anticancer effects. Di-O-caffeoylquinic acid, a well-documented natural product with known anti-inflammatory, antioxidative, and anticancer properties, could be a key contributor to the observed anticancer effects in our study [26]. Additionally, the presence of flavonoids, including quercetin and kaempferol, in APKH suggests their potential involvement in anticancer activities. The molecular structure of quercetin and kaempferol can be found in Supplementary Figure S5. These compounds, widely recognized for their anticancer potential, warrant further investigation to elucidate their precise contributions [27,28]. 

It is essential to consider the potential indirect effects of APKH on MYC expression and activity within DLBCL cells [29]. While our current data provide foundational insights, additional studies are essential to comprehensively elucidate these mechanisms. Specifically, given our data that APKH efficiently represses MYC expression at the transcriptional level, the hypothesis that APKH may modulate MYC expression through post-transcriptional mechanisms including translational mechanisms invites further investigation. 

Considering the possible effects on the translation of MYC transcripts, which might involve G-quadruplex and terminal oligopyrimidine (TOP) structures, we recognize the need for a targeted analysis of these structures. This could be achieved by examining the activity/phosphorylation of mTORC, S6K1, and 4EBP1, which are integral to the translation processes. Such studies could provide crucial insights into whether the protective effects of MYC overexpression via exogenous vectors are due to differences in transcript structures.

Thus, while this study lays the groundwork, subsequent research should focus on these specific molecular interactions to validate and potentially expand on the therapeutic implications of APKH in DLBCL treatment. Such investigations are imperative to move beyond in vitro analyses, paving the way for clinical applications that could significantly enhance patient outcomes.

In conclusion, our findings advocate for APKH as a promising natural component in the treatment of DLBCL, highlighting its ability to target the dysregulated MYC pathway and enhance chemotherapeutic efficacy [30,31,32]. While this study’s insights are derived from in vitro analyses, they lay a foundational basis for future in vivo research. Such studies are imperative to validate and extend our observations, ultimately facilitating the translation of APKH’s therapeutic potential into clinical applications. The exploration of APKH in combination with standard chemotherapy regimens opens new avenues for addressing the unmet needs in DLBCL treatment, marking a significant step forward in the quest for improved therapeutic strategies.

## Figures and Tables

**Table 1:** Molecular formulas and *m*/*z* values for the main compounds of APKH by UPLC Q-TOF/MS^e^ in the negative ionization mode.

No	RT (min)	MS^e^ Ion (*m*/*z*)	Molecular Formula	Compound
1	6.38	515.1186	C_25_H_23_O_12_	di-O-caffeoylquinic acid
2	8.71	301.0354, 285.0404	C_15_H_9_O_7_, C_15_H_9_O_6_	Flavonoids

## Data Availability

Data is contained within the article and Supplementary Materials.

## Supplementary Materials

The following supporting information can be downloaded at https://www.mdpi.com/article/10.3390/cimb46050278/s1. Table S1: Subtypes and genetic abnormalities in DLBCL; Table S2: Plant extracts with anti-DLBCL effect; Figure S1: APKH cytotoxicity in Ly1 vs. BM cells; Figure S2: Treatment with APKH did not reduce cell viability in U2932 cells; Figure S3: Ly1 DLBCL cells did not exhibit arrested cell cycle progression in response to APKH treatment; Figure S4: UPLC QTOF-MS analysis of APKH; Figure S5: The molecular structural of quercetin and kaempferol.
