# Supplementary material for: Ajania pacifica (Nakai) K. Bremer and Humphries Extract Limits MYC Expression to Induce Apoptosis in Diffuse Large B Cell Lymphoma
Source: Curr Issues Mol Biol. 2024 May 11;46(5):4580–94. doi: 10.3390/cimb46050278 (PMC11119827; doi:10.3390/cimb46050278)
Supplement: Supplementary file 1 [file cimb-46-00278-s001.zip › cimb-2889382-supplementary.pdf]

Supplementary Table S1

Subtypes and genetic abnormalities in DLBCL

Cell lines	Subtypes	Genetic Abnormalities
Ly1	GCB-DLBCL	BCL2 rearranged<br>BCL6 amplified<br>P53 mutated<br>MYC amplified
DHL6	GCB-DLBCL	BCL2 rearranged<br>AID, BCL6 amplified<br>P53 mutated<br>MYC translocated
U2932	ABC-DLBCL	BCL2 amplified<br>BCL6 amplified<br>MYC wild type

Supplementary Table S2

Plant extracts with anti-DLBCL effect

No.	Scientific name	Part used	Relative cell viability (%)	
			48h	72h
1	*Celtis choseniana Nakai*	Leaf, stem	99%	65%
2	*Eleocharis equisetiformis (Meinsh.) B.Fedtsch.*	Whole plant	89%	99%
3	*Populus nigra var. italica Koehne*	Whole plant	85%	76%
4	*Kerria japonica (L.) DC.*	Whole plant	100%	98%
5	*Iris pseudacorus L.*	Root	62%	76%
6	*Campsis grandiflora (Thunb.) K.Schum.*	Leaf	99%	100%
7	*Elaeagnus multiflora Thunb.*	Leaf, stem	96%	99%
8	*Diplazium subsinuatum (Wall. ex Hook. & Grev.) Taga wa*	Leaf	90%	100%
9	*Buddleja davidii Franch.*	Leaf, Branch	95%	98%
10	*Phyllostachys bambusoides Siebold & Zucc.*	Leaf, Branch	100%	93%
11	*Cimicifuga japonica (Thunb.) Spreng.*	Whole plant	91%	79%
12	*Galinsoga ciliata (Raf.) S.F.Blake*	Whole plant	97%	96%
13	*Ilex integra Thunb.*	Stem	100%	98%
14	*Lespedeza cuneata G.Don*	Top part	81%	63%
15	*Tilia taquetii C.K.Schneid.*	Leaf, Branch	85%	85%
16	*Euonymus japonicus 'Microphyllus'*	Beanch	95%	85%
17	*Prunus sibirica L.*	Branch	100%	100%
18	***Boehmeria pannosa Nakai & Satake ****	Leaf, Stem	66%	52%
19	*Akebia quinata (Houtt.) Decne.*	Leaf, Stem	100%	94%
20	*Celastrus stephanotiifolius Makino*	Branch	80%	61%
21	*Tritonia x crocosmiiflora G.Nicholson*	Whole plant	86%	73%
22	*Orixa japonica Thunb.*	Leaf	74%	67%
23	*Eurya emarginata (Thunb.) Makino*	Leaf	95%	61%
24	***Ajania pacifica (Nakai) K.Bremer & Humphries ****	Whole plant	67%	45%
25	*Strelitzia reginae Banks*	Top part	100%	88%
26	*Inula britannica var. japonica (Thunb.) Franch. & Sav.*	Whole plant	90%	87%
27	*Stellaria aquatica (L.) Scop.*	Whole plant	83%	98%
28	*Aspidistra elatior Blume*	Whole plant	98%	100%
29	*Agave americana L.*	Top part	99%	99%
30	*Zanthoxylum schinifolium Siebold & Zucc.*	Branch	89%	68%
31	*Ligustrum quihoui var. latifolium Nakai*	Branch	100%	100%
32	*Quercus glauca Thunb.*	Branch	100%	99%
33	*Justicia procumbens L.*	Whole plant	97%	86%
34	*Crataegus pinnatifida for. pilosa (C.K.Schneid.) Kitag.*	Branch	100%	93%
35	*Meliosma oldhamii Maxim.*	Leaf	98%	94%
36	*Salix caprea L.*	Branch	87%	89%
37	*Damnacanthus indicus C.F.Gaertn.*	Leaf, Branch	100%	98%
38	*Citrus tachibana (Makino) Yu. Tanaka*	Stem	100%	100%
39	*Hibiscus hamabo Siebold & Zucc.*	Leaf	100%	99%
40	*Taxus cuspidata var. latifolia (Pilg.) Nakai*	Branch	94%	86%

Supplementary Table S2

Plant extracts with anti-DLBCL effect

No.	Scientific name	Part used	Relative cell viability (%)	
			48h	72h
41	*Dracocephalum argunense Fisch. ex Link*	Whole plant	99%	100%
42	*Ficus erecta var. sieboldii (Miq.) King*	Branch	100%	100%
43	***Corydalis incisa (Thunb.) Pers. ****	Whole plant	45%	23%
44	*Semiaquilegia adoxoides (DC.) Makino*	Whole plant	97%	100%
45	*Stauntonia hexaphylla (Thunb.) Decne.*	Leaf, Stem	76%	71%
46	*Youngia japonica (L.) DC.*	Whole plant	95%	100%
47	*Torilis japonica (Houtt.) DC.*	Root	96%	100%
48	*Vitex rotundifolia L.f.*	Leaf, Stem	90%	90%
49	*Carpinus tschonoskii Maxim.*	Branch, Leaf	96%	89%
50	*Dryopteris crassirhizoma Nakai*	Top part	99%	99%
51	*Lagerstroemia indica L.*	Branch	98%	99%
52	*Acer triflorum Kom.*	Branch	98%	94%
53	*Sorbaria sorbifolia var. stellipila Maxim.*	Branch	97%	98%
54	*Rubus oldhamii Miq.*	Root	94%	84%
55	*Veronica persica Poir.*	Whole plant	99%	95%
56	*Malus baccata var. mandshurica (Maxim.) C.K.Schneid.*	Branch	100%	99%
57	***Fatsia japonica (Thunb.) Decne. & Planch. ****	Leaf, Stem	27%	46%
58	*Ligularia taquetii (H.Lev. & Vaniot) Nakai*	Whole plant	99%	100%
59	*Caryopteris divaricata (Siebold & Zucc.) Maxim.*	Top part	99%	97%
60	*Diplotaxis muralis DC.*	Whole plant	95%	98%
61	***Isodon inflexus (Thunb.) Kudo ****	Whole plant	38%	47%
62	*Carpinus laxiflora (Siebold & Zucc.) Blume*	Whole plant	78%	69%
63	*Lonicera praeflorens Batalin*	Branch	100%	99%
64	*Prunus speciosa (Koidz.) Nakai*	Branch	99%	98%
65	*Sisymbrium officinale (L.) Scop.*	Whole plant	97%	95%
66	*Leonurus japonicus Houtt.*	Root	100%	98%
67	*Larix kaempferi (Lamb.) Carriere*	Branch	63%	74%
68	*Artemisia fukudo Makino*	Whole plant	100%	98%
69	*Phryma leptostachya var. asiatica H. Hara*	Whole plant	100%	92%
70	*Photinia glabra (Thunb.) Maxim.*	Leaf, Stem	78%	86%
71	*Corylopsis gotoana var. coreana (Uyeki) T.Yamaz.*	Branch	89%	88%
72	***Ribes fasciculatum var. chinense Maxim. ****	Branch	56%	58%
73	*Metasequoia glyptostroboides Hu & W.C.Cheng*	Branch	76%	66%
74	*Arundo donax L.*	Top part	100%	100%
75	*Cercis chinensis Bunge*	Branch	93%	92%
76	*Rhynchosia volubilis Lour.*	Top part	99%	98%
77	*Spodiopogon sibiricus Trin.*	Whole plant	92%	80%
78	*Canavalia lineata (Thunb.) DC.*	Whole plant	99%	100%
79	*Ulmus laciniata (Trautv.) Mayr*	Leaf	96%	78%
80	*Fraxinus sieboldiana var. quadrijuga (Nakai) T.B.Lee*	Leaf	100%	86%

Supplementary Table S2

Plant extracts with anti-DLBCL effect

No.	Scientific name	Part used	Relative cell viability (%)	
			48h	72h
80	*Fraxinus sieboldiana var. quadrijuga (Nakai) T.B.Lee*	Leaf	100%	86%
81	*Artemisia montana (Nakai) Pamp.*	Whole plant	100%	100%
82	*Pennisetum alopecuroides (L.) Spreng.*	Whole plant	100%	100%
83	*Malus sieboldii (Regel) Rehder*	Branch	100%	96%
84	*Idesia polycarpa Maxim.*	Branch	77%	63%
85	*Boehmeria spicata (Thunb.) Thunb.*	Branch	97%	99%
86	*Prunus verecunda var. pendula (Nakai) W.T.Lee*	Branch	100%	97%
87	*Clinopodium chinense var. parviflorum (Kudo) H. Hara*	Whole plant	97%	96%
88	*Acer pictum subsp. mono (Maxim.) Ohashi*	Leaf	90%	92%
89	***Cornus macrophylla Wall. ****	Leaf	51%	52%
90	*Litsea japonica (Thunb.) Juss.*	Branch	56%	68%
91	*Sambucus sieboldiana var. pendula (Nakai) T.B.Lee*	Leaf	97%	82%
92	*Sorghum halepense for. muticum Hubb.*	Whole Plant	93%	86%
93	*Mirabilis jalapa L.*	Whole plant	96%	97%
94	*Aster arenarius (Kitam.) Nemoto*	Whole plant	99%	96%
95	*Euodia daniellii Hemsl.*	Leaf	99%	85%
96	*Osmanthus x fortune*	Leaf	88%	95%
97	*Ligustrum obtusifolium Siebold & Zucc.*	Leaf	91%	93%
98	*Ficus erecta Thunb.*	Branch	95%	100%
99	*Gardneria insularis Nakai*	Leaf	99%	100%
100	***Rudbeckia bicolor Nutt. ****	Seed	49%	48%

The data shown are the relative cell viability percent tested by Trypan blue assay.

* Potent anti-DLBCL activity against Ly1 cell line (<40% of control percentage 72h post-treatment.).

Supplementary Figure S1

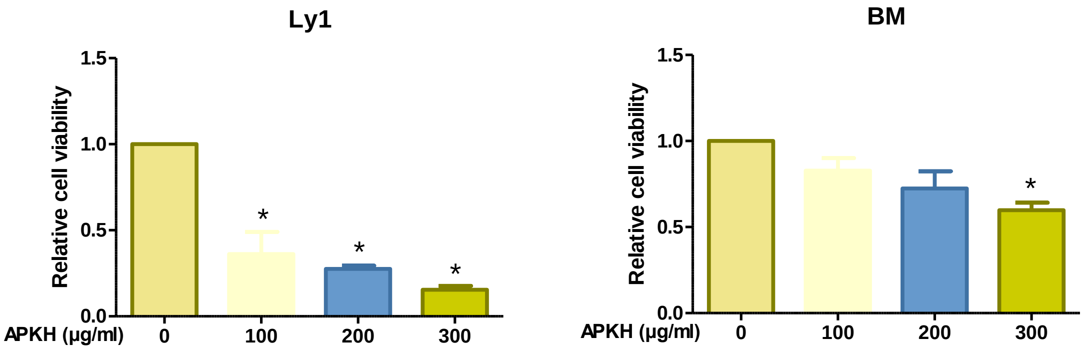

Supplementary Figure S1. APKH cytotoxicity in Ly1 vs. BM cells.

DLBCL Ly1 or normal bone marrow (BM) cells were exposed to APKH (0, 100, 200, or 300 μg/ml) for 24 h, and MTS assay was performed to measure cell viability. Statistical significance was analyzed using a two-tailed Student's t-test (*p < 0.05).

Supplementary Figure S2

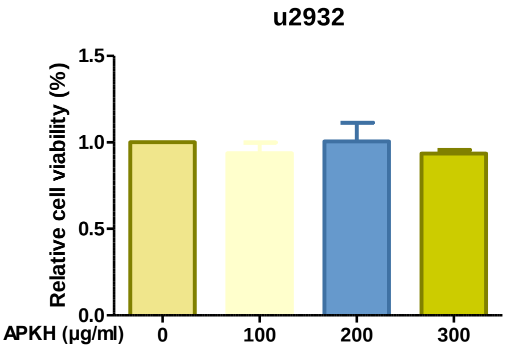

Supplementary Figure S2. Treatment with APKH did not reduce cell viability in U2932 cells.

U2932 cells were treated with APKH (0, 100, 200, or 300 μg/ml) for 24 h, and MTS assay was performed.

Supplementary Figure S3

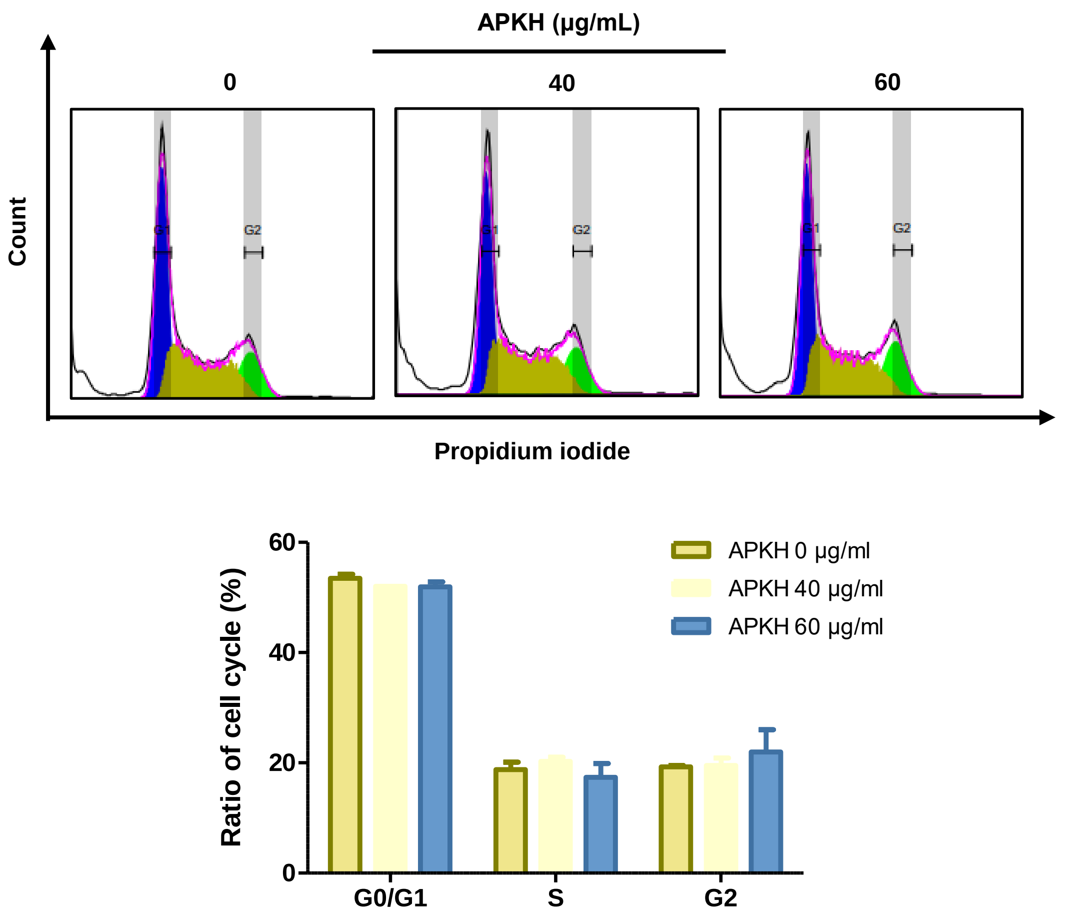

Supplementary Figure S3. Ly1 DLBCL cells did not exhibit arrested cell cycle progression in response to APKH treatment. Treatment of Ly1 cells with APKH for 24 h and analysis of cell cycle distribution by flow cytometry after staining with PI.

Supplementary Figure S4

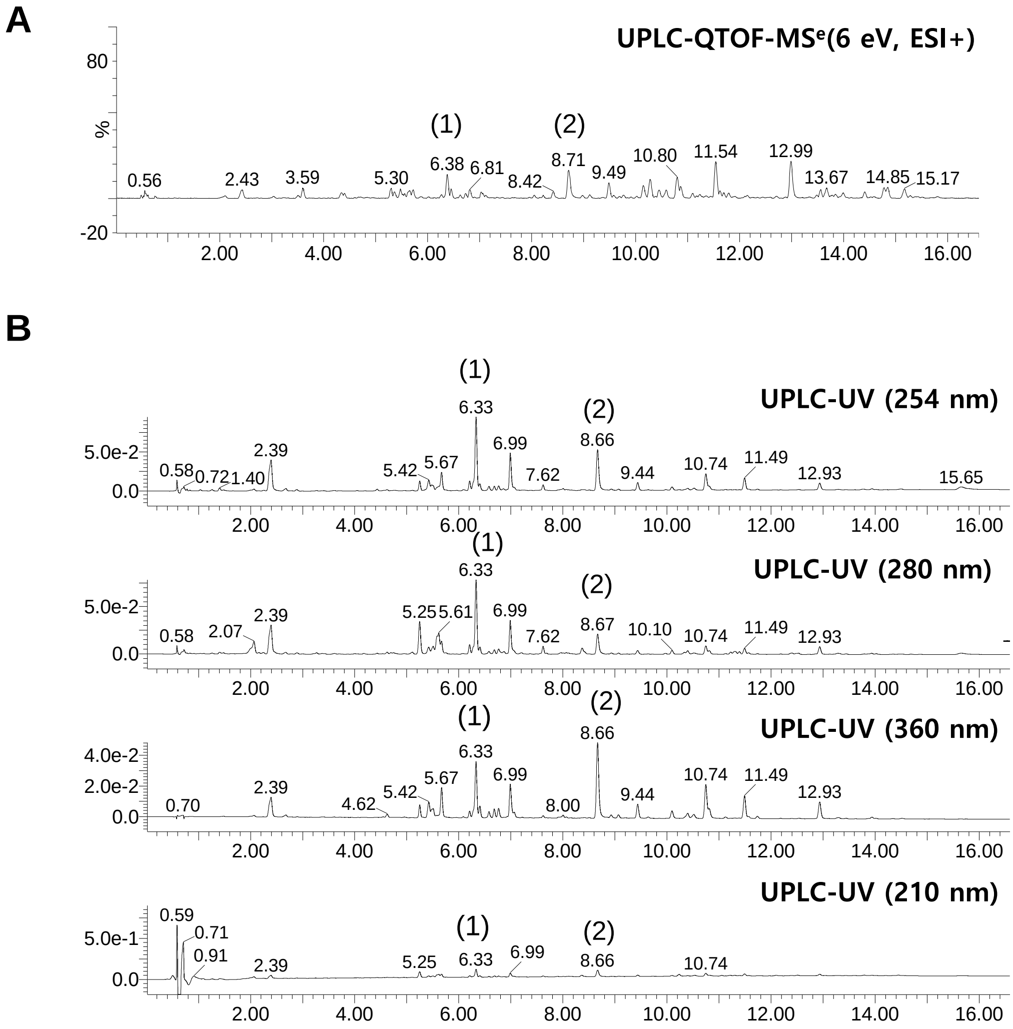

Supplementary Figure S4. UPLC QTOF-MS analysis of APKH.

(A) Positive ion mode. (B) Chromatographic profiles acquired under different UV wavelengths, including 254nm, 280nm, 360nm, and 210nm.

Supplementary Figure S5

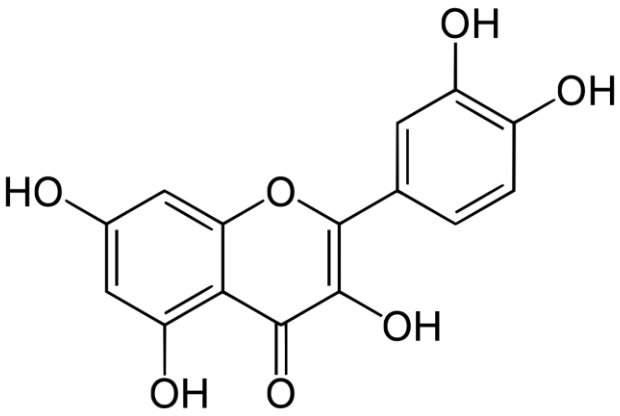

Quercetin

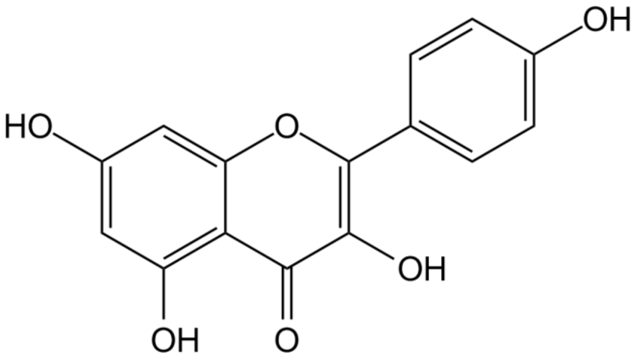

Kaempferol

Supplementary Figure S5. The molecular structure of quercetin and kaempferol.
